# Peroxisome Proliferator-Activated Receptors as Mediators of Phthalate-Induced Effects in the Male and Female Reproductive Tract: Epidemiological and Experimental Evidence

**DOI:** 10.1155/2008/359267

**Published:** 2007-12-06

**Authors:** Giuseppe Latini, Egeria Scoditti, Alberto Verrotti, Claudio De Felice, Marika Massaro

**Affiliations:** ^1^Division of Neonatology, Perrino Hospital, 72100 Brindisi, Italy; ^2^Clinical Physiology Institute (IFC-CNR), National Research Council of Italy, Lecce Section, 72100 Brindisi, Italy; ^3^Laboratory of General Physiology, Department of Biological and Environmental Sciences and Technology, University of Lecce, 73100 Lecce, Italy; ^4^Department of Medicine, Division of Pediatrics, University of Chieti, 66100 Chieti, Italy; ^5^Neonatal Intensive Care Unit, Azienda Ospedaliera-Universitaria Senese, 53100 Siena, Italy

## Abstract

There is growing evidence that male as well as female reproductive function has been declining in human and wildlife populations over the last 40 years. Several factors such as lifestyle or environmental xenobiotics other than genetic factors may play a role in determining adverse effects on reproductive health. Among the environmental xenobiotics phthalates, a family of man-made pollutants are suspected to interfere with the function of the endocrine system and therefore to be endocrine disruptors. The definition of endocrine disruption is today extended to broader endocrine regulations, and includes activation of metabolic sensors, such as the peroxisome proliferator-activated receptors (PPARs). Toxicological studies have shown that phthalates can activate a subset of PPARs. Here, we analyze the epidemiological and experimental evidence linking phthalate exposure to both PPAR activation and adverse effects on male and female reproductive health.

## 1. INTRODUCTION

The phthalate esters are a class of water-insoluble, high-production-volume, synthetic organic chemicals used widely in a variety of industrial applications, including personal-care products (e.g., perfumes, lotions, cosmetics), paints, and mainly as plasticizers to confer flexibility and durability to polyvinyl chloride- (PVC-) based plastics and to make the plastic appropriate to different uses, including food, construction industry, medical devices, and pharmaceuticals since about the 1930s [1–4]. However, these plasticizers are not chemically bound to the plastic products, but leak out from PVC items into the environment with time and use. As a consequence, they have been found everywhere in the environment and are universally considered ubiquitous environmental contaminants. Di-(2-ethylhexyl) phthalate (DEHP) is the most abundant phthalate in the environment and mono-(2-ethylhexyl) phthalate (MEHP) is its primary metabolite [1–4]. Other important phthalates production- and applicationwise are diethyl phthalate (DEP), dibutyl phthalate (DBP), di-iso- and di-n-butyl phthalate (DiBuP, DnBuP), butyl-benzyl phthalate (BBP), di-isononylphpthalate (DiNP) and di-n-octyl phthalate (DnOP) [5]. Humans are exposed to phthalates for their whole lifetime, since intrauterine life [6–11].

The ability of these pollutants to affect human health is a major concern. In particular, evidence suggestive of harmful effects on the male reproductive system and related outcomes have gradually accumulated in recent years. In addition, there is wide demonstration that reproductive functions are altered by endocrine disrupting chemicals (EDCs), including phthalates. These chemicals have been found to interfere with the function of the endocrine system, which is responsible for growth, sexual development, and many other essential physiological functions in both genders.

EDCs can act genomically, with agonistic or antagonistic effects on steroid receptors and may alter reproductive function and/or cause feminization by binding to oestrogen or androgen receptors. However, EDCs can also act by nongenomic mechanisms, altering steroid synthesis [12, 13].

The definition of endocrine disruption is today extended to broader endocrine regulations, and includes activation of metabolic sensors, such as a subset of nuclear hormone receptor superfamily members called peroxisome proliferator-activated receptors (PPARs).

To this regard, a large group of industrial and pharmaceutical chemicals, including phthalates, are known for their ability to provoke peroxisome proliferation, thus increasing both the size and number of peroxisomes [14]. Peroxisomes are essential organelles of eukaryotic origin, ubiquitously distributed in cells and organisms, which perform various metabolic functions (peroxide-derived respiration, beta oxidation of fatty acids, cholesterol metabolism, etc.) within the cell [15].

Many of the adaptive consequences for exposure to these pollutants are mediated by PPARs, members of the nuclear hormone receptor (NRs) superfamily of ligand-activated transcription factors. They are activated by binding of natural ligands, such as polyunsaturated fatty acids or by synthetic ligands. Three subtypes of PPARs (alpha, beta, and gamma) have been identified in different tissues, encoded by separate genes [16].

Several studies in recent years have revealed their importance in both normal physiology and in the pathology of various tissues [17, 18]. In particular, human and animal studies have demonstrated that PPARs are important in placental development [19], while they are believed to play an essential role in the adverse effects elicited by EDC [20].

The aim of this review is to explore how much evidence exists linking phthalate exposure, PPARs activation, and eventual actions of PPARs as mediators of environmental toxic substances for reproductive function in both genders.

## 2. ENVIRONMENTAL DISSEMINATION AND EPIDEMIOLOGICAL EVIDENCE OF PHTHALATE REPRODUCTIVE TOXICITY

Globally, more than 18 billion pounds of phthalates are used each year and well above two million tons of DEHP alone are produced annually worldwide [21]. Given their high production volume, common use, and widespread environmental contamination, humans are exposed to these compounds through ingestion, inhalation, and dermal exposures on a daily basis as testified by detection of phthalates in serum, seminal fluid, amniotic fluid, breast milk, and saliva [5, 9, 22–24]. These studies have provided evidence on the relatively high variation of phthalate exposure from day to day within individuals as well as between ethnic groups, geographic areas, and ages. In particular, general population can be exposed to DEHP to a much higher extent than previously believed and an exposure of children, twice as high as the exposure of adults with respect to their body weight, has been observed [23–26].

In particular, higher DEHP exposure has been documented in neonatal intensive-care-unit infants, because of multiple medical device-related DEHP exposure [27].

In addition, Blount et al. [28] found that women of reproductive age had significantly higher urinary levels of MBP (a reproductive and developmental toxicant in rodents) than other age/gender groups. However, in spite of the alarming wide environmental diffusion and use, studies in human populations suggesting an association between phthalate exposure and adverse reproductive health outcomes are limited yet.

To this regard, chronic occupational exposure to high levels of phthalates is associated with decreased rates of pregnancy and higher rates of miscarriage in female factory workers [29, 30]. Correspondently, higher urinary phthalate levels were observed to correlate with pregnancy complications such as anemia, toxemia, and pre-eclampsia in women living near a plastics manufacturer [31]. In addition, significantly high levels of phthalates were identified in girls with thelarche, suggesting an association between plasticizers with known estrogenic and antiandrogenic activity and the cause of premature breast development in a human female population [32].

In utero exposure to phthalates has been shown to be significantly associated with a shorter pregnancy duration [7, 8] and it has been hypothesized that phthalates may play a role in inducing and/or potentiating an intrauterine inflammatory response, a well established risk factor for prematurity [33]. Moreover, an association between phthalate exposure and endometriosis has been shown, suggesting a potential role for phthalate esters in the pathogenesis of this common cause of female infertility [34, 35]. More specifically to the male reproductive system, phthalate exposure seems to be tightly correlated to the impairment of androgen activity. For example, phthalate monoesters levels in breast milk resulted to be correlated with hormone levels in healthy boys, which were indicative of lower androgen activity and reduced Leydig cell function [36], and professional long-term exposure to phthalates has been reported to be associated with altered semen quality [37, 38] and decreased serum-free testosterone [39].

In addition, impaired testicular descent and decreased anogenital distance (AGD), the most sensitive marker of antiandrogen action in toxicological studies and a sensitive measure of prenatal antiandrogen exposure have been reported in boys whose mothers had elevated prenatal phthalate exposure [43]. All together, these findings suggest an impairment of sex hormone balance by prenatal and postnatal phthalate exposure but, although suggestive of the potentially dangerous effects of phthalate exposure on human health, they are not conclusive yet, and more epidemiologic data are needed in human populations along with a better mechanistic understanding of the phthalates activities. Although the possible mechanism of action by phthalates remains, to date, largely obscure, the use of animal models have enormously contributed to characterize the reproductive toxicity profiles of phthalates and to highlight the mechanisms possibly involved.

## 3. MALE AND FEMALE REPRODUCTIVE TRACT DEVELOPMENT: POSSIBLE INTERFERENCE SITE BY PHTHALATES

Male and female reproductive tract development is a dynamic process, requiring the production and the fine regulatory activity of sex steroid hormones: androgens, estrogens, and the progestagens [40]. Steroidal sex hormones regulate foetal developmental processes such as differentiation and sex determination. The major sites of synthesis of the sex steroids are corpus luteum for progestagens, testis for androgens, and ovaries for estrogens.

The biosynthesis of sex steroids is catalyzed by a series of enzymes that form the steroidogenic pathway [41]. This pathway causes the conversion of pregnenolone (cholesterol derivative key steroidogenic intermediate common to all classes of steroid hormones) to progesterone, the precursor for the testosterone that is formed in testis by Leydig cells through two ways: (1) Δ4-biosynthesis leads to progesterone, 17-*α*-hydroxyprogesterone, and androstenedione; (2) the Δ5-biosynthesis leads to 17-*α*-hydroxypregnenolone, dehydroepiandrosterone, and Δ5-androstendiol [41].

Androgens themselves can then be transformed to estrogens. The extent to which this biotransformation takes place depends on the expression of the various enzymes in specific tissues. The enzyme complex 19-hydroxylase-aromatase, which catalyzes the conversion of androgens to estrogens, plays a major role in this biotransformation [42].

The development of mammalian foetus into a male requires the production and action of steroid hormones, notably androgens and antimullerian hormone after testis formation, in contrast to the female development, a process largely hormone-independent [43].

Moreover, the mature reproductive function is under the regulation of the hypothalamus-pituitary-gonadal (HPG) axis. The limbic system of the brain releases specific neurotransmitters or neuropeptides that stimulate the hypothalamus to produce gonadotropin-releasing hormone (GnRH) which stimulates the pituitary gland to release specific hormones (gonadotrophins) that are transported via the blood stream to hormone-synthesizing tissues [44]. In the case of mammals, the gonadotrophins from the pituitary gland are luteinizing hormone (LH) and follicle-stimulating hormone (FSH). Under the influence of these substances, sex steroids, that is, estrogens and androgens, are released into the blood circulation from the ovaries and the testis, respectively. Negative feedback from the concentration of these gonadal steroids in the blood can lower or block the release of GnRH from the hypothalamus and of gonadotrophins at the pituitary level, thus modulating HPG axis [44].

**Figure 1:**
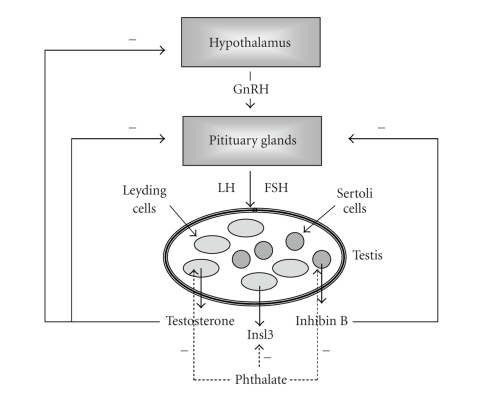


Keeping this in mind, it might be expected that any environmental, hormonally active chemicals capable of perturbing the adequate production and action of sex hormones or the balance between estrogens and androgens during foetal life have the potential to interfere with one or more critical aspects of reproductive function (Figure 1).

## 4. PRE- AND POSTNATAL DEVELOPMENTAL AND REPRODUCTIVE TOXICITY BY PHTHALATES

Chronic exposure of laboratory animals to phthalates has been reported to lead to severe adverse effects, including foetal death, carcinogenesis, teratogenesis, and hepatotoxicity [45–47]. In particular, a wide range of developmental and reproductive toxicities in mammals are induced by phthalates. Phthalates can directly affect fetal and neonatal testis differentiation, inducing male rat reproductive tract malformations, as well as testicular changes remarkably similar to testicular dysgenesis syndrome (TDS) in humans [48–52].

Testicular dysgenesis, or abnormal testicular development, after in utero phthalate exposure has been shown to be associated with abnormal function of both Sertoli and Leydig cells and abnormal sex organs development [52, 53].

Sertoli cells play a critical role in foetal testis development regulating the dynamic process of movement, organization, differentiation of all the cell types within the testis [54]. As a consequence, the abnormal function of Sertoli cells associated with phthalate exposure [52, 53] might alter the differentiation signals normally implicated in tissue morphogenesis, thus leading to many of the histological and functional anomalies observed in TDS (Figure 1).

Leydig cells, the principal providers of steroid hormones in the testis, are also targeted by phthalates. To this regard, the highly conserved role of testosterone and dihydrotestosterone (DHT), in driving male reproductive tract development (masculinization) is well known. As a consequence, in rodents the whole period of male genital tract differentiation is particularly susceptible to the effects of antiandrogens, as demonstrated by in utero exposure to flutamide, (a well-known androgen receptor antagonist) and phthalates both inducing abnormalities of androgen-regulated sexual differentiation [49]. In addition, the administration of synthetic estrogens, such as diethylstilboestrol (DES), to pregnant women and rodents causes reproductive tract abnormalities in the offspring, including cryptorchidism, [55] as well as a dose-dependent reduction in the number of Sertoli cells critically involved in spermatogenesis [56]. The ability of estrogens to reduce androgen levels or expression of androgen receptor is relevant [57]. These results suggest that abnormal intrauterine hormone levels with decreased androgen production/action or increased estrogens levels may play a role in determining adverse effects on reproductive health. Correspondently, critical to the induction of phthalate testicular toxicity is the considerable reduction in fetal and postnatal testosterone levels observed after in utero exposure to phthalates at the critical window for the androgen-dependent reproductive tract development [49, 52, 53, 58]. In particular, the exposure to DEHP decreases testosterone to levels similar to those normally found in females leading to incomplete masculinization and hypospadias and cryptorchidism [58]. Thus, several phthalate esters have been shown to carry out “antiandrogenic” activity through a mechanism that is distinct from androgen-receptor antagonism, that is, targeting the Leydig cells testosterone biosynthesis machinery. In addition, genes directly associated with testosterone biosynthesis are uniformly downregulated by phthalate exposure in the fetal testis [59]. These steroidogenic genes include those involved in cholesterol handling, such as scavenger receptor class B type 1 (SR-B1) implicated in the selective cholesterol esters uptake from high density lipoproteins, steroidogenic acute regulatory protein (StAR), that mediates cholesterol transport across the mitochondrial membrane, the rate limiting enzyme in testosterone biosynthesis, that is, cholesterol side-chain cleavage enzyme (P450 scc), that converts cholesterol into pregnenolone, 3*β*-hydroxysteroid dehydrogenase (3 *β*HSD), and CYP17*α* [59, 60]. In addition, phthalates alter the expression of genes encoding sex steroid metabolizing enzymes in the gonads and peripheral organs such as the liver. Among these, 5*α*-reductase, that converts testosterone to DHT, was upregulated by DEHP in the prepubertal rat testis [61]. Aside from the interference with steroid synthesis and metabolism, the induction of cryptorchidism by phthalates is mediated by the alternative mechanism acting at the initial hormone-independent phase of testicular descent. Phthalates have indeed been shown to alter the expression of insulinlike hormone 3 (Insl3) in fetal Leydig cells [62], which plays a role in guiding the testis during its first phase of transabdominal descent.

In postnatal exposure, a strong species difference in the phthalate responsiveness is evident, with some species (Syrian hamsters, e.g.,) more resistant to phthalate toxicity possibly as a consequence of an inefficient metabolic transformation of diesters to monoesters [63]. Younger animals result, in general, more sensitive than adult ones [64]. For example, Grey observed a decrease in seminiferous tubule diameter in testis and accessory sex organs (seminal vesicle and prostate) weight after phthalate exposure in 4-week-old, but not in 15-week-old rats [64]. These effects were associated with the induction of apoptosis in germ cells, likely as a consequence of an increased generation of oxidative stress and concomitant alteration of antioxidant defences by phthalate [65]. Correspondently, the FSH signalling pathway for Sertoli cell proliferation and differentiation resulted to be impaired after phthalate exposure [66, 67].

Also in postnatal and adult rats phthalates affected steroid hormone synthesis and metabolism, as indicated by decreased testosterone serum levels in male rats acutely exposed to some active phthalates and by a decreased testosterone secretion by cultured Leydig cells treated with MEHP [68]. However, contrasting results were observed by Akingbemi et al. [69] and Eagon et al. [70] in male rat chronically exposed to environmentally relevant low levels of DEHP. Increased LH and testosterone serum levels together with an increased serum estrogen likely due to impaired Leydig cell steroidogenesis and compensatory Leydig cell proliferation were observed. The modulation by phthalate of many estrogen metabolizing enzymes seems to be very complex, since it has been reported both a downregulation [71, 72] and an upregulation [73] of the aromatase gene after phthalate exposure, depending on the cell type analyzed.

Overall, the data presented here demonstrated that certain phthalates like other environmental chemicals are capable of disrupting male reproductive tract organogenesis and function when administered to laboratory animals during pregnancy and/or postnatal life, producing types of malformations and histological changes causing infertility remarkably similar to those observed in human TDS. One mechanism responsible for this effects may be the ability to disrupt the endocrine balance, that is, androgen/estrogen activities, essential for reproductive system development and homeostasis, acting as environmental antiandrogen compounds [74]. Although this raises concern towards other factors such as lifestyle that might have influenced human fertility [75].

## 5. THE PPAR SYSTEM AT THE CROSSROADS BETWEEN METABOLISM AND REPRODUCTION

The identification of phthalates as environmental chemicals belonging to the family of peroxisome proliferators (PP) has shed new insight into the potential molecular mechanism of phthalate action in the reproductive system of mammals. The pleiotropic effects induced by PP including phthalates in the rodent liver are mediated by the activation of PPARs, ligand-activated transcription factors belonging to the nuclear receptor superfamily, which also includes the steroid and thyroid hormone receptors [76]. Thus far, three PPAR isoforms (*α*, *β*, or *δ*, and *γ*), encoded by separate genes, have been identified in various tissues, with PPAR*α* predominantly expressed in the liver, PPAR*γ* in adipose tissue, and PPAR*β* in a wider range of tissue [16]. Upon activation by their lipophilic ligands, PPARs regulate gene transcription by binding to PPAR response elements (PPRE) within the promoter of target genes as heterodimers with retinoic X receptors (RXR) [16, 77]. PPARs can also repress gene expression in a DNA-binding-dependent way through the recruitment of corepressors to unliganded PPARs as well as in a DNA-binding-independent manner by interfering with other nuclear signalling pathways via protein-protein interaction (leading to formation of inactive complexes) or via competition for limiting amounts of the heterodimerization partner RXR or coactivators [78]. Fatty acids and eicosanoids have been identified as natural ligands for PPARs. More potent synthetic PPAR ligands include the fibrate and thiazolidinedione drugs, clinically used as hypolipidemic and antidiabetic agents, respectively. Since the discovery of PPARs in 1990 [17], several functions have been attributed to these receptors. PPARs play critical physiological roles regulating lipid and glucose homeostasis, cellular differentiation, proliferation, and the inflammatory/immune response, with subsequent clinically relevant implication in several diseases including dyslipidemia, diabetes, cancer, atherosclerosis. PPAR*α* has been demonstrated to play a role in regulating lipid catabolism, whereas PPAR*γ* controls adipocyte differentiation and lipid storage [16, 77]. Although PPAR*β* is less well understood, it might be a mediator in the control of brain lipid metabolism, fatty acid-induced adipogenesis, and atherogenic inflammation [77]. Given the extensive crosstalk between PPARs and other transcription factors and signalling events regulating energy balance, differentiation and other significant physiological processes in many tissues, the involvement of environmental chemicals in the PPAR system may potentially result in pathophysiologically relevant consequences for human health.

The role of PPAR*α* in PP-induced hepatic proliferative responses was established by the development of PPAR*α*-deficient mice by Lee et al. [79]. In contrast to wild-type control animals, PPAR*α* homozygous-deficient mice do not exhibit hepatic peroxisomal proliferation in response to treatment with PP. Aside from modest changes in lipid profile and weight, PPAR*α*-deficient mice are otherwise phenotypically normal [80]. Thus, the major hepatic effects of PP, including hepatocarcinogenic effects, are mediated by PPAR*α*-dependent gene transcription and signalling events. The response to PP seems to be species-specific, with rats and mice being quite sensitive to them and humans, guinea pigs, and other species being refractory [80]. Remarkably, the hepatotoxic effects of PP are lost in humans due to the lower level of PPAR*α* expression in human liver than in rodent one [81] and to species-specific responsiveness of PPAR*α* [82].

Before focusing on the potential involvement of PPARs in the reproductive effects of phthalate, it would be useful to consider PPAR expression pattern in the reproductive system, since the potential PPAR-mediated effects of phthalates depend on tissue distribution of the PPAR isoforms and the PPAR-responsive genes in each tissue. All PPAR isoforms are expressed in the central nervous system and in reproductive tissues, such as gonads (testis and ovary), uterus, prostate, mammary gland, pituitary gland [83]. In the testis, both somatic and germ cells express PPAR isoforms: PPAR*α* and *β* are expressed in Leydig cells and cells of seminiferous tubule (Sertoli cells and germ cells) [60, 84], while PPAR*γ* seems to be only detectable in Sertoli cells, although weak PPAR*γ* expression in germ cells has recently been reported [85]. All PPAR isoforms have been detected in the ovary [84]. PPAR*γ* is the predominant isoform expressed in the granulosa cells and preovulatory follicles, but its expression falls after the LH surge [86]. In addition, PPAR*γ* is less strongly expressed in the techal cells and in corpus luteum where it increases after ovulation [86]. However, in the absence of fertilization or embryo implantation, PPAR*γ* expression decreases as a result of corpus luteum regression [87]. Finally, PPAR*γ* is expressed in uterine tissue, blastocyst and, together with PPAR*α* and *β*, in gestational tissues [88, 89].

The physiological role of PPARs in the reproductive tissues is not completely understood but while, on one hand, PPAR*α*-null mice remain viable and fertile [79], on the other hand, PPAR*β* deletion impairs fertility [90] and PPAR*γ*-null mutation is even embryonically lethal [91]. Indeed, recent findings suggested putative important roles for PPARs in reproductive system: the ability of PPARs to regulate energy balance may represent a potential molecular link between reproductive function and glucose and lipid metabolism. It has been shown that PPAR*α*, whose expression is upregulated by FSH in cultured seminiferous tubules [92], may affect spermatozoa fertility by promoting lipid storage mobilization and modifying phospholipid composition. PPAR*β* seems to play an important role in embryo implantation as showed by its strong upregulation during the decidualization process and the appearance of placental malformations in PPAR*β*-null mice [90]. Finally, several lines of evidence suggest that PPAR*γ* is critically involved in follicular development, ovulation, maintenance of corpus luteum during pregnancy, and maturation and function of placenta [83].

## 6. MECHANISM OF PHTHALATE ESTER REPRODUCTIVE TOXICITY: POTENTIAL ROLE OF PPARS

The involvement of phthalate-PPAR interactions in the reproductive biology alteration derives from recent findings demonstrating that phthalates are able to activate PPARα and PPARγ isoforms. Metabolic conversion of diesters to the hydrolytic monoesters seems to be essential to obtain PPAR activation and toxicological effects [93]. Indeed, hepatic peroxisomal proliferation and the associated hepatocarcinogenic response induced in rodents by DEPH are mediated by its bioactive metabolite MEHP [94], which is able to activate both human and rodent PPAR*α* and PPAR*γ* in in vitro transactivation assay [95]. In addition to MEHP, other structurally diverse phthalate monoesters, most notably monobenzyl phthalate (mBzP), the primary metabolite of butyl benzyl phthalate (BBP), and mono-*sec*-butyl phthalate (MBuP) are capable of activating both human PPAR isoforms and target genes [93, 96] with potential implication for human health as these reproductive toxicants have been detected in human urine samples at exceptionally higher levels than MEHP itself [28]. However, it has been recently found that the diesters DEHP and BBP themselves were able to activate PPAR*α* and PPAR*γ* to some extent, although it was likely attributable to low level of esterases activity in the cell model used [96]. Interestingly, analyses of structure-activity relationship have found that PP in general are amphipathic carboxilates thus resembling natural PPAR ligands such as long-chain saturated and unsaturated fatty acids [97]. The carboxyl moiety of monoesters is critical for ligand activity: for example, some DEHP metabolites, such as MEHP and 2-ethylhexanoic acid, are more potent PPAR activators than 2-etylhexanol metabolite [98]. The rank order for phthalate activation of mouse and human PPAR*α* and PPAR*γ* agrees with the relative ability of phthalate esters to induce the classical PPAR responses, that are liver peroxisomal proliferation in rodents for PPAR*α* and adipocyte differentiation for PPAR*γ* [93, 99]. Indeed, it has been found that esters with long and branch-side chain are more potent PPAR activators than those containing short-chains or straight-chains. As regards PPAR*β*, only phthalate monoesters with longer and branch-side chains can activate this isoform but at a concentration higher than that required for activation of PPAR*α* and PPAR*γ* [100]. Importantly, human PPARs are less sensitive to phthalate monoesters than the corresponding mouse receptors [93]. Since the activation of PPAR assessed by transactivation assay might result from indirect events, such as endogenous production of a metabolite from the test compound or release of endogenous ligand, these compounds had to be tested further for direct binding to the PPARs. Although activation of PPARs by some phthalates may occur indirectly through release of endogenous lipid activators (fatty acids) from carrier proteins, notably fatty acid binding protein (FABP) or through a yet unidentified intermediate factor [101], recent findings reported that some relevant monoester phthalates are able of directly binding PPAR*α* and PPAR*γ* receptors [96]. Consistent with their ability to activate PPARs in transactivation assay, BBP and DBP weakly interact with both isoforms.

Although in most cases there has been found a correlation between PPAR activation by phthalate monoesters and reproductive toxicity by the corresponding diesters, there exist also findings weakening the assumption of a general obligatory role for PPARs in mediating phthalate-induced reproductive effects. For example, while di-isononyl phthalate (DINP) is a weak reproductive toxicant [102], its monoester metabolite MINP is a moderately strong PPAR activator [100]. In addition, DBP is a strong reproductive toxicant through its proximal metabolite MBP [103] and induces hepatotoxicity in rodents via PPAR*α* [104], although MBP only weakly activates PPARs in transactivation assay [93]. One possible interpretation of these discordant results may be the involvement of an indirect mechanism of PPAR activation mediated by an unknown endogenous metabolite activator, not necessarily detectable by using transactivation assay.

Only a few studies in PPAR*α*-null mice directly determined the role of PPAR in phthalate-induced male developmental and reproductive toxicities. The study by Peters et al. [105] showed that prenatal exposure to DEHP caused developmental malformations in both wild-type and PPAR*α* knockout mice, thus suggesting a PPAR*α*-independent mechanism. However, it is difficult to draw any conclusion about the role of PPAR*α* in phthalate reproductive toxicity since the intrauterine administration of DEHP occurred before the critical period of reproductive tract differentiation. Another important animal study demonstrated that intrauterine DEHP-treated PPAR*α*-deficient mice, predominantly normal at earlier time point, developed delayed testicular, renal and developmental toxicities, but not liver toxicity, compared to wild types [104], thus first confirming the early observation by Lee et al. about the PPAR*α* dependence of liver response and, more importantly, indicating that DEHP may induce reproductive toxicity through both PPAR*α*-dependent and -independent mechanism. Another study found that the administration of DEHP resulted in milder testis lesions and higher testosterone levels in PPAR*α*-null mice than in wild-type mice [106]. In contrast, the PPAR*α*-independent reproductive toxicity observed by Ward et al. may conceivably be mediated by other PPAR isoforms, such as PPAR*β* and PPAR*γ*, or by a nonreceptor-mediated organ-specific mechanism. Unfortunately, till now no studies have been performed in PPAR*β*-null mice, and the toxicological impacts of phthalates that activate PPAR*γ* are unknown. Determining a role for PPAR*γ* in phthalate-induced reproductive toxicity requires testis-specific-knockout mice as PPAR*γ* deletion results in the death of the embryo [91]. Notably, both PPAR*α* and PPAR*γ* are responsive to DEHP in vitro and are translocated to the nucleus in primary Sertoli cells after incubation of these cells with phthalate esters [107, 108]. Given the key role played by Sertoli cells in driving testis morphogenesis, it may be therefore hypothesized that the impairment of this cell type by MEHP contributed to the observed testicular toxicity.

The potential of PPARs to mediate the endocrine disruption activity by phthalates is also suggested from the finding that a few genes involved in steroid biosynthesis and metabolism are directly regulated by PPARs. MEHP activates both PPAR*α* and PPAR*γ* in cultured rat granulosa cells which cause a complete inhibition of aromatase gene expression [109–111]. In addition, the estradiol metabolizing enzyme 17*β*-HSD IV has been shown to be induced by MEHP in the liver and granulosa cells through a PPAR*α*-dependent mechanism [112]. Therefore, both decreased estradiol synthesis and increased estradiol metabolism contribute to suppressed serum estradiol levels observed after DEHP in vivo exposure and to the subsequent female reproductive toxicity [71, 72, 113]. Finally, the induction by DEHP of FABP expression in the liver via PPAR*α* [114] and in granulosa cells via both PPAR*α* and PPAR*γ* [115] may play important role in the mechanism of phthalate effect on steroid hormones since FABP functions as an intracellular gateway for PPAR agonists [116] and as a donor of potential fatty acid ligands of PPARs [101].

Taking into account the specific tissue distribution and the physiological roles of PPAR isoforms, one could speculate upon some phthalate effects in mammals. It is known that cells exposed to PP undergo oxidative stress possibly due to PPAR*α*-mediated activation of metabolizing enzymes in the liver and associated with the hepatic toxicity of DEHP [117]. Genes involved in oxidative stress response have been shown to be upregulated in the liver by DEHP exposure [118]. In addition, the induction of xenobiotic metabolizing enzymes by PPAR*α* after DEHP exposure could increase the susceptibility to other environmental toxicants requiring metabolic activation [118]. PPAR*γ* is a prototypic adipocyte differentiation regulator [119] and activation of PPAR*γ* by phthalates in other tissue and subsequent alteration of differentiation pathways may be implicated in phthalate teratogenic effects. In addition, PPAR*γ* may be part of the LH-induced luteinization in the ovary since its activation causes aromatase downregulation, this event being essential for the postovulatory phenotype [120]. The activation of PPAR*γ* by phthalates in the preovulatory follicle prevented the estradiol increase necessary for stimulating the ovulatory surge of LH and prematurely induces follicle differentiation to a postovulatory phenotype [113].

## 7. DEVELOPMENTAL AND REPRODUCTIVE TOXICITY OF PHTHALATES IN FEMALE ANIMAL MODELS

The above-mentioned epidemiological evidence suggesting adverse consequences for female reproductive function [30, 31] stimulated more in depth studies in animal models on the issue. Besides causing developmental toxicity, including high incidence of foetus death and malformations and reduced foetal body weight, DEHP administration to pregnant rodents decreased embryo implantation and increased resorptions [121, 122]. These effects were mimicked by other phthalate esters thus representing both male and female reproductive toxicants in rodents [123].

The administration of phthalate esters, including DEHP and its metabolite MEHP, to adult female rats caused an increase in the estrous cycle length and dysovulation, associated with polycystic ovaries, and decreased serum levels of estradiol [71]. These functional changes were associated with morphological alteration of the preovulatory follicle, the site of estradiol production, where granulosa cells were smaller in DEHP-treated mice than in control rats, and incapable of mounting an ovulatory surge of LH. Regarding the molecular mechanism by which DEHP/MEHP suppressed estradiol production in the granulosa cells, it has been found that MEHP inhibits FSH-stimulated cAMP accumulation and progesterone production in granulosa cells [124]. When the progesterone precursor pregnenolone is added to granulosa cell cultures treated with MEHP, the inhibition of progesterone production is reversed [125]. However MEHP did not decrease the expression of P450 scc [126], the major regulatory site of progesterone production by cAMP which converts cholesterol to pregnenolone [127]. In addition to reducing progesterone production at a site prior to pregnenolone, MEHP also reduces estradiol production by affecting aromatase gene expression, the rate-limiting enzyme that converts testosterone to estradiol. Aromatase is stimulated by FSH-mediated pathways and techal androgens. Androgens are the substrates for aromatization to estradiol in granulosa cells [128]. Thus, MEHP is able to decrease estradiol production independent of its effect on FSH–cAMP and decreases aromatase activity without acting as a direct enzyme inhibitor [72]. Furthermore, the induction by both DEHP and DBP of the estradiol metabolizing enzyme 17*β*-HSD IV in the liver and granulosa cells [112, 129] contributes to explain the suppressed serum estradiol levels after DEHP exposure and the significant increase in serum levels of estrone, the primary metabolite of estradiol, observed in DBP-treated rats [71].

Overall, these findings underline once again that phthalate toxicant effects on female reproductive system is attributable to an interference with the complex and tightly regulated machinery involved in steroid synthesis and metabolism. Notably, the pathways leading to production of ovarian hormones are similar in rodent models and humans, and using the rodent model to determine the mechanism of action of MEHP will aid in understanding how exposure to this chemical may affect ovarian function in women.

## 8. CONCLUSIONS

Phthalates are environmental contaminants with significant human exposures. These chemicals may act as EDCs and alter reproductive function and/or cause feminization raising concern about the potential health hazards posed by such exposures. The adverse effects of phthalates have been chiefly studied in animal models, while their potential toxicity to humans together with the possible involvement of PPARs in mediating these effects on the reproductive health has to be more properly evaluated. Pre- and/or perinatal periods appear to be critical windows of exposure, because of their high sensitivity to hormonal dysregulation by EDCs. Thus, the acquisition of more detailed data on human exposure during these time periods is essential. It has been proposed that impairment of reproductive development and function in both genders by phthalates relates to abnormal steroid biosynthesis and metabolism and seems to be at least in part mediated by the activation of the PPAR signalling pathway. Molecular basis for the adverse health effects proposed to be associated with human phthalate exposure have to be elucidated. Finally, analysis of the effects of phthalate exposures on gonadotropin and steroid hormone levels should form part of overall risk assessment in human populations.

## Figures and Tables

**Table 1:** Structures and related name of the most common phthalate monoesters. Diesters of *o*-phthalic acid are quickly metabolized in vivo to their active metabolites, the monesters. The length and structure of the side chain are important for toxicity.

Chemical structure	Systematic name	Abbreviation
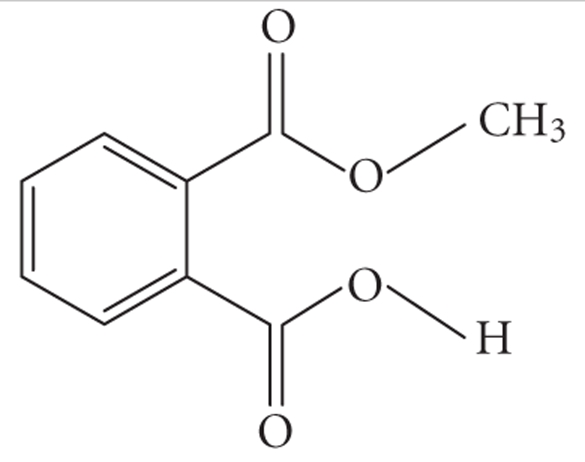	Monomethyl phthalate	MMP

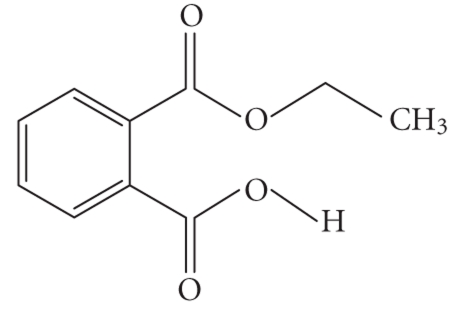	Monoethyl phthalate	MEP

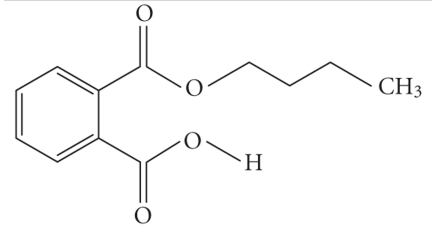	Monobutyl phthalate	MBP

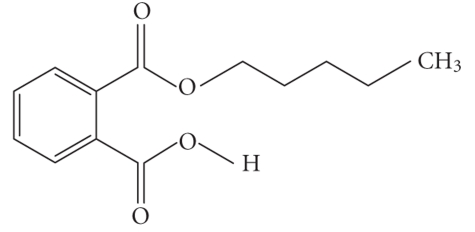	Monopentyl phthalate	MPP

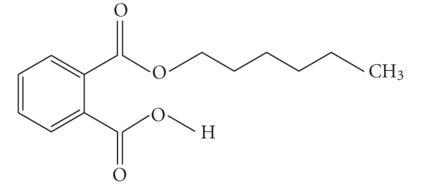	Monohexyl phthalate	MHP

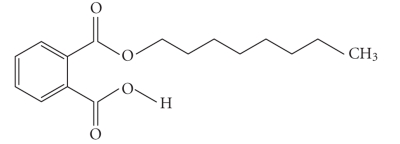	Monopropyl phthalate	MPrP

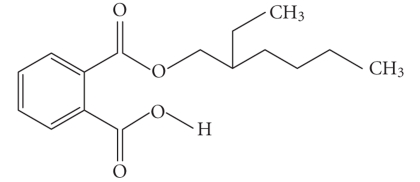	Mono-(2-ethylhexyl) phthalate	MEPH

## References

[1] ATDSR (1995). Toxicological Profile for Diethylphthalate.

[2] ATDSR (1997). Toxicological Profile for di-n-octyl phthalate.

[3] ATDSR (2001). Toxicological Profile for di-n-butyl phthalate.

[4] ATDSR (2002). Toxicological Profile for di-(2-ethylhexyl)phthalate (DEHP).

[5] Koch HM, Rossbach B, Drexler H, Angerer J (2003). Internal exposure of the general population to DEHP and other phthalates—determination of secondary and primary phthalate monoester metabolites in urine. *Environmental Research*.

[6] Adibi JJ, Perera FP, Jedrychowski W (2003). Prenatal exposures to phthalates among women in New York and Krakow, Poland. *Environmental Health Perspectives*.

[7] Latini G, De Felice C, Presta G (2003). In utero exposure to di-(2-ethylhexyl)phthalate and duration of human pregnancy. *Environmental Health Perspectives*.

[8] Latini G, de Felice C, Presta G (2003). Exposure to di-(2-ethylhexyl)phthalate in humans during pregnancy: a preliminary report. *Biology of the Neonate*.

[9] Silva MJ, Reidy JA, Herbert AR, Preau JL, Needham LL, Calafat AM (2004). Detection of phthalate metabolites in human amniotic fluid. *Bulletin of Environmental Contamination and Toxicology*.

[10] Swan SH (2006). Prenatal phthalate exposure and anogenital distance in male infants. *Environmental Health Perspectives*.

[11] Swan SH, Main KM, Liu F (2005). Decrease in anogenital distance among male infants with prenatal phthalate exposure. *Environmental Health Perspectives*.

[12] Kavlock R, Boekelheide K, Chapin R (2002). NTP center for the evaluation of risks to human reproduction: phthalates expert panel report on the reproductive and developmental toxicity of di-n-octyl phthalate. *Reproductive Toxicology*.

[13] Waring RH, Harris RM (2005). Endocrine disrupters: a human risk?. *Molecular and Cellular Endocrinology*.

[14] Ehrmann J, Vavrusová N, Collan Y, Kolár Z (2002). Peroxisome proliferator-activated receptors (PPARs) in health and disease. *Biomedical Papers of the Medical Faculty of the University Palacky*.

[15] Tabak HF, Hoepfner D, Zand AVD, Geuze HJ, Braakman I, Huynen MA (2006). Formation of peroxisomes: present and past. *Biochimica et Biophysica Acta*.

[16] Lemberger T, Desvergne B, Wahli W (1996). Peroxisome proliferator-activated receptors: a nuclear receptor signaling pathway in lipid physiology. *Annual Review of Cell and Developmental Biology*.

[17] Issemann I, Green S (1990). Activation of a member of the steroid hormone receptor superfamily by peroxisome proliferators. *Nature*.

[18] Yu S, Reddy JK (2007). Transcription coactivators for peroxisome proliferator-activated receptors. *Biochimica et Biophysica Acta*.

[19] Fournier T, Tsatsaris V, Handschuh K, Evain-Brion D (2007). PPARs and the placenta. *Placenta*.

[20] Feige JN, Gelman L, Rossi D (2007). The endocrine disruptor monoethyl-hexyl-phthalate is a selective peroxisome proliferator-activated receptor γ
modulator that promotes adipogenesis. *Journal of Biological Chemistry*.

[21] Blount BC, Milgram KE, Silva MJ (2000). Quantitative detection of eight phthalate metabolites in human urine using HPLC-APCI-MS/MS. *Analytical Chemistry*.

[22] Calafat AM, Needham LL, Silva MJ, Lambert G (2004). Exposure to di-(2-ethylhexyl)phthalate among premature neonates in a neonatal intensive care unit. *Pediatrics*.

[23] Silva MJ, Barr DB, Reidy JA (2004). Urinary levels of seven phthalate metabolites in the U.S. population from the National Health and Nutrition Examination Survey (NHANES) 1999-2000. *Environmental Health Perspectives*.

[24] Silva MJ, Slakman AR, Reidy JA (2004). Analysis of human urine for fifteen phthalate metabolites using automated solid-phase extraction. *Journal of Chromatography B*.

[25] Koch HM, Bolt HM, Angerer J (2004). Di-(2-ethylhexyl)phthalate (DEHP) metabolites in human urine and serum after a single oral dose of deuterium-labelled DEHP. *Archives of Toxicology*.

[26] Koch HM, Drexler H, Angerer J (2004). Internal exposure of nursery-school children and their parents and teachers to di-(2-ethylhexyl)phthalate (DEHP). *International Journal of Hygiene and Environmental Health*.

[27] Green R, Hauser R, Calafat AM (2005). Use of di-(2-ethylhexyl)phthalate-containing medical products and urinary levels of mono-(2-ethylhexyl)phthalate in neonatal intensive care unit infants. *Environmental Health Perspectives*.

[28] Blount BC, Silva MJ, Caudill SP (2000). Levels of seven urinary phthalate metabolites in a human reference population. *Environmental Health Perspectives*.

[29] Milkov LE, Aldyreva MV, Popova TB (1973). Health status of workers exposed to phthalate plasticizers in the manufacture of artificial leather and films based on PVC resins. *Environmental Health Perspectives*.

[30] Aldyreva MV, Klimova TS, Izyumova AS, Timofievskaya LA (1975). The effect of phthalate plasticizers on the generative function. *Gigiena Truda I Professional'nye Zabolevaniia*.

[31] Tabacova S, Little R, Balabaeva L (1999). Maternal exposure to phthalates and complications of pregnancy. *Epidemiology*.

[32] Colón I, Caro D, Bourdony CJ, Rosario O (2000). Identification of phthalate esters in the serum of young Puerto Rican girls with premature breast development. *Environmental Health Perspectives*.

[33] Gonçalves LF, Chaiworapongsa T, Romero R (2002). Intrauterine infection and prematurity. *Mental Retardation and Developmental Disabilities Research Reviews*.

[34] Cobellis L, Latini G, De Felice C (2003). High plasma concentrations of di-(2-ethylhexyl)phthalate in women with endometriosis. *Human Reproduction*.

[35] Reddy BS, Rozati R, Reddy BVR, Raman NVVSS (2006). Association of phthalate esters with endometriosis in Indian women. *BJOG: An International Journal of Obstetrics and Gynaecology*.

[36] Lottrup G, Andersson A-M, Leffers H (2006). Possible impact of phthalates on infant reproductive health. *International Journal of Andrology*.

[37] Duty SM, Silva MJ, Barr DB (2003). Phthalate exposure and human parameters. *Epidemiology*.

[38] Rozati R, Reddy PP, Reddanna P, Mujtaba R (2002). Role of environmental estrogens in the deterioration of male factor fertility. *Fertility and Sterility*.

[39] Pan G, Hanaoka T, Yoshimura M (2006). Decreased serum free testosterone in workers exposed to high levels of di-n-butyl phthalate (DBP) and di-2-ethylhexyl phthalate (DEHP): a cross-sectional study in China. *Environmental Health Perspectives*.

[40] Jost A (1960). Action of various sex and related steroids on the growth and sexual differentiation of fetuses. *Acta Endocrinologica. Supplementum*.

[41] Brooks RV (1975). Androgens. *Clinical Endocrinology & Metabolism*.

[42] Suhara K, Ohashi K, Takeda K, Katagiri M (1986). P-450(11β)-dependent conversion of androgen to estrogen, the aromatase reaction. *Biochemical and Biophysical Research Communications*.

[43] Wilson JD (1978). Sexual differentiation. *Annual Review of Physiology*.

[44] Rodgers CH (1975). Neuroendocrine mechanisms responsible for gonadotropin release. *The Journal of Reproductive Medicine*.

[45] Lake BG (1995). Mechanisms of hepatocarcinogenicity of peroxisome-proliferating drugs and chemicals. *Annual Review of Pharmacology and Toxicology*.

[46] Latini G, Massaro M, De Felice C (2005). Prenatal exposure to phthalates and intrauterine inflammation: a unifying hypothesis. *Toxicological Sciences*.

[47] Lock EA, Mitchell AM, Elcombe CR (1989). Biochemical mechanisms of induction of hepatic peroxisome proliferation. *Annual Review of Pharmacology and Toxicology*.

[48] Mylchreest E, Cattley RC, Foster PMD (1998). Male reproductive tract malformations in rats following gestational and lactational exposure to di-(n-butyl) phthalate: an antiandrogenic mechanism?. *Toxicological Sciences*.

[49] Mylchreest E, Sar M, Cattley RC, Foster PMD (1999). Disruption of androgen-regulated male reproductive development by di-(n-butyl) phthalate during late gestation in rats is different from flutamide. *Toxicology and Applied Pharmacology*.

[50] Mylchreest E, Sar M, Wallace DG, Foster PMD (2002). Fetal testosterone insufficiency and abnormal proliferation of Leydig cells and gonocytes in rats exposed to di-(n-butyl) phthalate. *Reproductive Toxicology*.

[51] Mylchreest E, Wallace DG, Cattley RC, Foster PMD (2000). Dose-dependent alterations in androgen-regulated male reproductive development in rats exposed to di-(n-butyl) phthalate during late gestation. *Toxicological Sciences*.

[52] Foster PMD (2005). Mode of action: impaired fetal Leydig cell function—effects on male reproductive development produced by certain phthalate esters. *Critical Reviews in Toxicology*.

[53] Foster PMD (2006). Disruption of reproductive development in male rat offspring following in utero exposure to phthalate esters. *International Journal of Andrology*.

[54] Koopman P (2001). Gonad development: signals for sex. *Current Biology*.

[55] Stillman RJ (1982). In utero exposure to diethylstilbestrol: adverse effects on the reproductive tract and reproductive performance in male and female offspring. *American Journal of Obstetrics and Gynecology*.

[56] Sharpe RM, McKinnell C, Kivlin C, Fisher JS (2003). Proliferation and functional maturation of Sertoli cells, and their relevance to disorders of testis function in adulthood. *Reproduction*.

[57] Rivas A, Fisher JS, McKinnell C, Atanassova N, Sharpe RM (2002). Induction of reproductive tract developmental abnormalities in the male rat by lowering androgen production or action in combination with a low dose of diethylstilbestrol: evidence for importance of the androgen-estrogen balance. *Endocrinology*.

[58] Parks LG, Ostby JS, Lambright CR (2000). The plasticizer di-ethylhexyl phthalate induces malformations by decreasing fetal testosterone synthesis during sexual differentiation in the male rat. *Toxicological Sciences*.

[59] Barlow NJ, Phillips SL, Wallace DG, Sar M, Gaido KW, Foster PMD (2003). Quantitative changes in gene expression in fetal rat testes following exposure to di-(n-butyl) phthalate. *Toxicological Sciences*.

[60] Shultz VD, Phillips S, Sar M, Foster PMD, Gaido KW (2001). Altered gene profiles in fetal rat testes after in utero exposure to di-(n-butyl) phthalate. *Toxicological Sciences*.

[61] Kim H-S, Saito K, Ishizuka M, Kazusaka A, Fujita S (2003). Short period exposure to di-(2-ethylhexyl)phthalate regulates testosterone metabolism in testis of prepubertal rats. *Archives of Toxicology*.

[62] Wilson VS, Lambright C, Furr J (2004). Phthalate ester-induced gubernacular lesions are associated with reduced insl3 gene expression in the fetal rat testis. *Toxicology Letters*.

[63] Gray TJB, Rowland IR, Foster PMD, Gangolli SD (1982). Species differences in the testicular toxicity of phthalate esters. *Toxicology Letters*.

[64] Gray TJB, Gangolli SD (1986). Aspects of the testicular toxicity of phthalate esters. *Environmental Health Perspectives*.

[65] Agarwal A, Saleh RA, Bedaiwy MA (2003). Role of reactive oxygen species in the pathophysiology of human reproduction. *Fertility and Sterility*.

[66] Li L-H, Jester WF, Laslett AL, Orth JM (2000). A single dose of di-(2-ethylhexyl)phthalate in neonatal rats alters gonocytes, reduces Sertoli cell proliferation, and decreases cyclin D2 expression. *Toxicology and Applied Pharmacology*.

[67] Li L-H, Jester WF, Orth JM (1998). Effects of relatively low levels of mono-(2-ethylhexyl)phthalate on cocultured Sertoli cells and gonocytes from neonatal rats. *Toxicology and Applied Pharmacology*.

[68] Jones HB, Garside DA, Liu R, Roberts JC (1993). The influence of phthalate esters on Leydig cell structure and function in vitro and in vivo. *Experimental and Molecular Pathology*.

[69] Akingbemi BT, Youker RT, Sottas CM (2001). Modulation of rat Leydig cell steroidogenic function by di-(2-ethylhexyl)phthalate. *Biology of Reproduction*.

[70] Eagon PK, Chandar N, Epley MJ, Elm MS, Brady EP, Rao KN (1994). Di-(2-ethylhexyl)phthalate-induced changes in liver estrogen metabolism and hyperplasia. *International Journal of Cancer*.

[71] Davis BJ, Maronpot RR, Heindel JJ (1994). Di-(2-ethylhexyl)phthalate suppresses estradiol and ovulation in cycling rats. *Toxicology and Applied Pharmacology*.

[72] Davis BJ, Weaver R, Gaines LJ, Heindel JJ (1994). Mono-(2-ethylhexyl)phthalate suppresses estradiol production independent of FSH-cAMP stimulation in rat granulosa cells. *Toxicology and Applied Pharmacology*.

[73] Akingbemi BT, Ge R, Klinefelter GR, Zirkin BR, Hardy MP (2004). Phthalate-induced Leydig cell hyperplasia is associated with multiple endocrine disturbances. *Proceedings of the National Academy of Sciences of the United States of America*.

[74] Fisher JS (2004). Environmental anti-androgens and male reproductive health: focus on phthalates and testicular dysgenesis syndrome. *Reproduction*.

[75] Sharpe RM, Franks S (2002). Environment, lifestyle and infertility—an inter-generational issue. *Nature Cell Biology*.

[76] Desvergne B, Wahli W (1999). Peroxisome proliferator-activated receptor: nuclear control of metabolism. *Endocrine Reviews*.

[77] Bocher V, Chinetti G, Fruchart JC, Staels B (2002). Role of the peroxisome proliferator-activated receptors (PPARS) in the regulation of lipids and inflammation control. *Journal of Biomedicine and Biotechnology*.

[78] Chinetti G, Fruchart J-C, Staels B (2000). Peroxisome proliferator-activated receptors (PPARs): nuclear receptors at the crossroads between lipid metabolism and inflammation. *Inflammation Research*.

[79] Lee SS, Pineau T, Drago J (1995). Targeted disruption of the α isoform of the peroxisome proliferator-activated receptor gene in mice results in abolishment of the pleiotropic effects of peroxisome proliferators. *Molecular and Cellular Biology*.

[80] Gonzalez FJ, Peters JM, Cattley RC (1998). Mechanism of action of the nongenotoxic peroxisome proliferators: role of the peroxisome proliferator-activated receptor. *Journal of the National Cancer Institute*.

[81] Palmer CN, Hsu MH, Griffin KJ, Raucy JL, Johnson EF (1998). Peroxisome proliferator activated receptor-α expression in human liver. *Molecular Pharmacology*.

[82] Keller H, Devchand PR, Perroud M, Wahli W (1997). PPARα structure-function relationships derived from species-specific differences in responsiveness to hypolipidemic agents. *Biological Chemistry*.

[83] Froment P, Gizard F, Defever D, Staels B, Dupont J, Monget P (2006). Peroxisome proliferator-activated receptors in reproductive tissues: from gametogenesis to parturition. *Journal of Endocrinology*.

[84] Braissant O, Foufelle F, Scotto C, Dauca M, Wahli W (1996). Differential expression of peroxisome proliferator-activated receptors (PPARs): tissue distribution of PPAR-α, -β, and -γ in the adult rat. *Endocrinology*.

[85] Thomas K, Sung D, Chen X, Gibbs R, McCarrey J, Walker W Developmental patterns of PPAR/RXR gene expression during spermatogenesis.

[86] Komar CM, Braissant O, Wahli W, Curry TE (2001). Expression and localization of PPARs in the rat ovary during follicular development and the periovulatory period. *Endocrinology*.

[87] Viergutz T, Loehrke B, Poehland R, Becker F, Kanitz W (2000). Relationship between different stages of the corpus luteum and the expression of the peroxisome proliferator-activated receptor γ protein in bovine large lutein cells. *Journal of Reproduction and Fertility*.

[88] Mohan M, Ryder S, Claypool PL, Geisert RD, Malayer JR (2002). Analysis of gene expression in the bovine blastocyst produced in vitro using suppression-subtractive hybridization. *Biology of Reproduction*.

[89] Berry EBE, Eykholt R, Helliwell RJA, Gilmour RS, Mitchell MD, Marvin KW (2003). Peroxisome proliferator-activated receptor isoform expression changes in human gestational tissues with labor at term. *Molecular Pharmacology*.

[90] Barak Y, Liao D, He W (2002). Effects of peroxisome proliferator-activated receptor δ
on placentation, adiposity, and colorectal cancer. *Proceedings of the National Academy of Sciences of the United States of America*.

[91] Barak Y, Nelson MC, Ong ES (1999). PPARγ is required for placental, cardiac, and adipose tissue development. *Molecular Cell*.

[92] Schultz R, Yan W, Toppari J, Völkl A, Gustafsson J-Å, Pelto-Huikko M (1999). Expression of peroxisome proliferator-activated receptor α messenger ribonucleic acid and protein in human and rat testis. *Endocrinology*.

[93] Hurst CH, Waxman DJ (2003). Activation of PPARα and PPARγ by environmental phthalate monoesters. *Toxicological Sciences*.

[94] Albro PW, Lavenhar SR (1989). Metabolism of di-(2-ethylhexyl)phthalate. *Drug Metabolism Reviews*.

[95] Maloney EK, Waxman DJ (1999). Trans-activation of PPARα and PPARγ by structurally diverse environmental chemicals. *Toxicology and Applied Pharmacology*.

[96] Lapinskas PJ, Brown S, Leesnitzer LM (2005). Role of PPARα in mediating the effects of phthalates and metabolites in the liver. *Toxicology*.

[97] Kliewer SA, Sundseth SS, Jones SA (1997). Fatty acids and eicosanoids regulate gene expression through direct interactions with peroxisome proliferator-activated receptors α and γ. *Proceedings of the National Academy of Sciences of the United States of America*.

[98] Keith Y, Cornu MC, Canning PM, Foster JMD, Lhuguenot JC, Elcombe CR (1992). Peroxisome proliferation due to di-(2-ethylhexyl) adipate, 2-ethylhexanol and 2-ethylhexanoic acid. *Archives of Toxicology*.

[99] Barber ED, Astill BD, Moran EJ (1987). Peroxisome induction studies on seven phthalate esters. *Toxicology and Industrial Health*.

[100] Bility MT, Thompson JT, McKee RH (2004). Activation of mouse and human peroxisome proliferator-activated receptors (PPARs) by phthalate monoesters. *Toxicological Sciences*.

[101] Luebker DJ, Hansen KJ, Bass NM, Butenhoff JL, Seacat AM (2002). Interactions of flurochemicals with rat liver fatty acid-binding protein. *Toxicology*.

[102] Gray LE, Ostby J, Furr J, Price M, Veeramachaneni DNR, Parks L (2000). Perinatal exposure to the phthalates DEHP, BBP, and DINP, but not DEP, DMP, or DOTP, alters sexual differentiation of the male rat. *Toxicological Sciences*.

[103] Foster PMD, Cattley RC, Mylchreest E (2000). Effects of di-n-butyl phthalate (DBP) on male reproductive development in the rat: implications for human risk assessment. *Food and Chemical Toxicology*.

[104] Ward JM, Peters JM, Perella CM, Gonzalez FJ (1998). Receptor and nonreceptor-mediated organ-specific toxicity of di-(2-ethylhexyl)phthalate (DEHP) in peroxisome proliferator-activated receptor alpha-null mice. *Toxicology and Pathology*.

[105] Peters JM, Taubeneck MW, Keen CL, Gonzalez FJ (1997). Di-(2-ethylhexyl)phthalate induces a functional zinc deficiency during pregnancy and teratogenesis that is independent of peroxisome proliferator-activated receptor-α. *Teratology*.

[106] Gazouli M, Yao Z-X, Boujrad N, Corton JC, Culty M, Papadopoulos V (2002). Effect of peroxisome proliferators on Leydig cell peripheral-type benzodiazepine receptor gene expression, hormone-stimulated cholesterol transport, and steroidogenesis: role of the peroxisome proliferator-activator receptor α. *Endocrinology*.

[107] Dufour JM, Vo M-N, Bhattacharya N, Okita J, Okita R, Kim KH (2003). Peroxisome proliferators disrupt retinoic acid receptor alpha signaling in the testis. *Biology of Reproduction*.

[108] Bhattacharya N, Dufour JM, Vo M-N, Okita J, Okita R, Kwan HK (2005). Differential effects of phthalates on the testis and the liver. *Biology of Reproduction*.

[109] Mu Y-M, Yanase T, Nishi Y, Takayanagi R, Goto K, Nawata H (2001). Combined treatment with specific ligands for PPARγ:RXR nuclear receptor system markedly inhibits the expression of cytochrome P450arom in human granulosa cancer cells. *Molecular and Cellular Endocrinology*.

[110] Lovekamp-Swan T, Chaffin CL (2005). The peroxisome proliferator-activated receptor γ ligand troglitazone induces apoptosis and p53 in rat granulosa cells. *Molecular and Cellular Endocrinology*.

[111] Corton JC, Lapinskas PJ (2005). Peroxisome proliferator-activated receptors: mediators of phthalate ester-induced effects in the male reproductive tract?. *Toxicological Sciences*.

[112] Corton JC, Bocos C, Moreno ES, Merritt A, Cattley RC, Gustafsson J-Å (1997). Peroxisome proliferators alter the expression of estrogen-metabolizing enzymes. *Biochimie*.

[113] Lovekamp-Swan T, Davis BJ (2003). Mechanisms of phthalate ester toxicity in the female reproductive system. *Environmental Health Perspectives*.

[114] Poirier H, Niot I, Monnot M-C (2001). Differential involvement of peroxisome-proliferator-activated receptors α and δ in fibrate and fatty-acid-mediated inductions of the gene encoding liver fatty-acid-binding protein in the liver and the small intestine. *Biochemical Journal*.

[115] Lovekamp-Swan T, Jetten AM, Davis BJ (2003). Dual activation of PPARα and PPARγ by mono-(2-ethylhexyl)phthalate in rat ovarian granulosa cells. *Molecular and Cellular Endocrinology*.

[116] Wolfrum C, Borrmann CM, Börchers T, Spener F (2001). Fatty acids and hypolipidemic drugs regulate peroxisome proliferator-activated receptors α- and γ-mediated gene expression via liver fatty acid binding protein: a signaling path to the nucleus. *Proceedings of the National Academy of Sciences of the United States of America*.

[117] Reddy JK, Rao MS (1989). Oxidative DNA damage caused by persistent peroxisome proliferation: its role in hepatocarcinogenesis. *Mutation Research*.

[118] Wong JS, Gill SS (2002). Gene expression changes induced in mouse liver by di-(2-ethylhexyl)phthalate. *Toxicology and Applied Pharmacology*.

[119] Tontonoz P, Hu E, Graves RA, Budavari AI, Spiegelman BM (1994). mPPARγ2: tissue-specific regulator of an adipocyte enhancer. *Genes and Development*.

[120] Fitzpatrick SL, Carlone DL, Robker RL, Richards JS (1997). Expression of aromatase in the ovary: down-regulation of mRNA by the ovulatory luteinizing hormone surge. *Steroids*.

[121] Kaul AF, Souney PF, Osathanondh R (1982). A review of possible toxicity of di-2-ethylhexylphthalate (DEHP) in plastic intravenous containers: effects on reproduction. *Drug Intelligence and Clinical Pharmacy*.

[122] Tomita I, Nakamura Y, Yagi Y, Tutikawa K (1986). Fetotoxic effects of mono-2-ethylhexyl phthalate (MEHP) in mice. *Environmental Health Perspectives*.

[123] Heindel JJ, Powell CJ (1992). Phthalate ester effects on rat Sertoli cell function in vitro: effects of phthalate side chain and age of animal. *Toxicology and Applied Pharmacology*.

[124] Treinen KA, Dodson WC, Heindel JJ (1990). Inhibition of FSH-stimulated cAMP accumulation and progesterone production by mono-(2-ethylhexyl)phthalate in rat granulosa cell cultures. *Toxicology and Applied Pharmacology*.

[125] Treinen KA, Heindel JJ (1992). Evidence that MEHP inhibits rat granulosa cell function by a protein kinase C-independent mechanism. *Reproductive Toxicology*.

[126] Lovekamp TN, Davis BJ (2001). Mono-(2-ethylhexyl)phthalate suppresses aromatase transcript levels and estradiol production in cultured rat granulosa cells. *Toxicology and Applied Pharmacology*.

[127] Hsueh AJ, Adashi EY, Jones PB, Welsh TH (1984). Hormonal regulation of the differentiation of cultured ovarian granulosa cells. *Endocrine Reviews*.

[128] Richards JS (1980). Maturation of ovarian follicles: actions and interactions of pituitary and ovarian hormones on follicular cell differentiation. *Physiological Reviews*.

[129] Fan L-Q, Cattley RC, Corton JC (1998). Tissue-specific induction of 1β7-hydroxysteroid dehydrogenase type IV by peroxisome proliferator chemicals is dependent on the peroxisome proliferator-activated receptor α. *Journal of Endocrinology*.

